# The Influence of the Early Childhood Education and Care Environment on Young Children’s Physical Activity: Development and Reliability of the PLAYCE Study Environmental Audit and Educator Survey

**DOI:** 10.3390/ijerph17072497

**Published:** 2020-04-06

**Authors:** Clover Maitland, Leanne Lester, Stewart G. Trost, Michael Rosenberg, Jasper Schipperijn, Georgina Trapp, Pulan Bai, Hayley Christian

**Affiliations:** 1 School of Human Sciences, The University of Western Australia, Perth, WA 6009, Australia; clover.maitland@uwa.edu.au (C.M.); leanne.lester@uwa.edu.au (L.L.); michael.rosenberg@uwa.edu.au (M.R.); 2 Institute of Health and Biomedical Innovation at Queensland Centre for Children’s Health Research, School of Exercise and Nutrition Science, Queensland University of Technology, Brisbane, QLD 4001, Australia; s.trost@qut.edu.au; 3 Department of Sports Science and Clinical Biomechanics, University of Southern Denmark, Odense, DK-5230, Denmark; jschipperijn@health.sdu.dk; 4 School of Population and Global Health, The University of Western Australia, Perth, WA 6009, Australia or or; 5 Telethon Kids Institute, University of Western Australia, Nedlands, WA 6009, Australia

**Keywords:** preschooler, childcare, physical environment, policy environment, educator practices, social-ecological model

## Abstract

(1) Background: Participation in physical activity is crucial for the healthy growth and development of young children. More robust measurement of environmental influences on children’s physical activity in early childhood education and care (ECEC) settings may help resolve inconsistencies in the literature. This study evaluated the reliability of an environmental audit and educator practice survey for assessing ECEC physical, policy, and social environments related to young children’s physical activity. (2) Methods: A convenience sample of 20 ECEC centres participated in this PLAYCE (Play Spaces and Environments for Children’s Physical Activity) sub-study. Trained auditors conducted audits and educators completed surveys, two weeks apart. Test-retest reliability of the survey (*n* = 32), inter-rater (*n* = 20 pairs) and intra-rater reliability (*n* = 38) of the audit was assessed using intra-class correlation coefficients (ICCs), Kappa statistics and percent agreement. (3) Results: Intra-rater and inter-rater reliability ICCs for outdoor equipment, spaces and features were good to excellent (ICC = 0.70–0.94), while ratings for indoor equipment, media and spaces varied from fair to excellent (ICC = 0.46–0.78). The majority of items were rated by Kappa as moderate or above for intra-rater, inter-rater and survey test-retest reliability. (4) Conclusions: The PLAYCE Study instruments provide reliable measures of ECEC physical activity environments which can help to better understand influences on young children’s physical activity.

## 1. Introduction

Physical activity is vital for the healthy growth and development of young children [1]. For pre-school aged children (1–5 years), participation in regular physical activity is associated with better health and developmental outcomes including a healthy weight status, fitness, motor development and bone health [2,3]. However, many preschool children do not meet recommendations for physical activity, although estimates vary considerably [4].

The majority of young children in Europe aged 3–4 years, over half in Australia aged 2–3 years, and almost one quarter of US children under 5 years of age, attend some type of formal early childhood education and care (ECEC) [5,6,7]. Overall, a large proportion of young children spend a significant amount of time in ECEC. ECECs play an important role in promoting and providing physical activity opportunities for young children enabling them to meet the recommended 180 minutes of physical activity per day [8,9]. There is evidence that ECEC environments have a greater influence on preschooler’s physical activity than socio-demographic factors [10]. Thus, the ECEC setting is an ideal target for further research and intervention to increase preschoolers’ physical activity.

In line with the socio-ecological framework [11], a range of amenable environmental factors have been positively associated with preschoolers’ physical activity whilst attending ECEC [12,13]. Physical environmental factors include the availability of play equipment and size of play space [14,15,16,17,18], and social environmental factors include educator practices such as providing children with opportunities to be active and participating in physical activity education and training [19,20,21]. A 2016 review of the correlates of children’s physical activity and sedentary behaviour in ECECs found the most significant influence within the ECEC setting was the outdoor physical environment [12]. However, inconsistencies exist as it also concluded that there is inconclusive or limited evidence for most social, physical and policy environmental factors, except for educators providing active opportunities for children and the size of play space [12]. This may be attributed, in part, to varied and imprecise measurement. Therefore valid and reliable measures of the ECEC social, physical and policy environment have been recommended [22].

Robust assessment of ECEC environments is important both for determining influences on young children’s physical activity, and the monitoring and assessment of ECEC regulatory standards [23]. Of the various environmental measurement instruments available, the Environment and Policy Assessment and Observation (EPAO) tool has been most extensively used to assess the social, physical and policy environment of ECEC services [24]. While US and northern European studies show associations between EPAO sub-scales and physical activity [19,25], recent studies in the UK and Australia found no relationship [26,27], raising concerns about applicability across different ECEC environments. Furthermore, aspects of the natural environment, the availability of electronic media, and other features of the indoor and outdoor space, may all potentially influence physical activity for children in ECEC [28]. Measurement of these aspects of the environment may be better addressed in instruments that assess ECEC environments for physical activity.

The purpose of this study was to develop and test the reliability of two measurement instruments—an environmental audit and an educator practice survey—for assessing the ECEC physical, policy and social environment related to young children’s physical activity.

## 2. Materials and Methods

This research was part of the PLAYCE (Play Spaces and Environments for Children’s Physical Activity) study which sought to determine features of the ECEC environment associated with preschoolers’ physical activity and inform ECEC regulatory standards in Australia. This cross-sectional observational study collected accelerometry data from 1596 children aged 2–5 years attending ECEC in Perth, Western Australia. Potential individual, social and physical environmental influences on children’s physical activity at the ECEC, home and neighborhood level, were collected using a parent survey and geographic information systems measures, in addition to the instruments described in this manuscript. The study methodology has been published previously [28].

### 2.1. Ethical Consideration

Ethics approval was granted from The University of Western Australia Human Research Ethics Committee (approval number: RA/4/1/7417).

### 2.2. Instrument Development

The PLAYCE Study ECEC environmental audit and educator practice survey were informed by a review of measurement tools used to measure the correlates of physical activity and sedentary behaviour of children attending ECEC and findings from previous pilot work [29]. Established items with good validity and reliability in international contexts were chosen where possible and adapted to suit the Australian ECEC environment if necessary. New items were drafted in consultation with the research team [28]. Each instrument was pilot tested by six people in three ECEC centres. The environmental audit was completed by six research project team members who provided feedback on the study protocol, including practical implementation, and identified items that were unclear or missing. The educator practice survey was completed by six ECEC educators who provided written feedback on the relevance and clarity of questions. Feedback was collated and both instruments were revised and finalised in discussion with study investigators.

PLAYCE ECEC environmental audit—the audit was designed to objectively assess elements of the physical and policy environment of ECEC centres that may support (or hinder) children’s physical activity. The physical environmental component assessed the indoor and outdoor environment including spaces, equipment, and built and natural features and included: (1) outdoor physical activity equipment (number, type and condition of portable and fixed); (2) outdoor play environment (number and type of play spaces, natural features, paths); (3) indoor physical activity equipment (number, type and condition of portable and fixed); (4) indoor media equipment (number and type of portable and fixed); and (5) indoor play environment (presence and connection of play spaces). The policy environmental component of the audit addressed physical activity, screen time and sun-protection policy. The majority of items were modified from previous instruments [17,30,31,32] for the Australian ECEC environment (Table 1 footnotes).

PLAYCE ECEC educator practice survey—the survey assessed the practices of ECEC staff to promote and support children’s physical activity including: (1) time provided for physical activity (duration and frequency of indoor and outdoor physical activity); (2) educator role in physical activity (lead, supervise, encourage, join in); (3) educator physical activity practices (incorporate in other activities, use as behaviour management, talk about it, use promotional materials); (4) educator professional development; (5) provision of physical activity equipment (availability and offering); and (6) indoor spaces for play (accessibility, availability and use). In relation to sedentary behaviour the survey assessed: (7) time provided for screen time (duration and opportunity for TV time and interactive screen time; use as a reward). All items were taken from the Nutrition and Physical Activity Self-Assessment for Child Care (Go NAP SAAC) instruments [31], except availability and use of indoor play space items [30] and new items (Table 2). Most items were updated and modified for the Australian ECEC context; for example, using the term ‘educators’ instead of teachers, referring to the Australian specific ‘Get up and Grow’ physical activity resources, separating indoor and outdoor physical activity frequency, and including interactive whiteboards as an educational screen time example.

### 2.3. Study Participants and Protocol

Data were collected to assess intra-rater reliability and inter-rater reliability for the PLAYCE ECEC environmental audit and test-retest reliability for the PLAYCE ECEC educator practice survey. The PLAYCE principal investigator and project coordinator briefed a team of ten auditors, consisting of PLAYCE investigators, project staff and postgraduate students on how to administer the environmental audit. The 90-minute briefing introduced the instruments, providing examples and considerations for completing, and presented the research protocol for conducting the environmental audit in ECECs. A convenience sample of 12 ECEC centres in the Perth metropolitan area (Western Australia) were invited, and eight provided written consent to participate.

To assess the intra-rater reliability of the PLAYCE ECEC environmental audit, each centre was visited at Time 1 by between three and nine auditors who completed the physical environmental sections by walking through each indoor room and outdoor space and recording their answers. Auditors accessed a copy of the centre policies to complete the policy environmental sections. Two weeks later the same auditors returned to the centre and conducted the audit a second time using the same protocol (Time 2). Auditors completed the audit independently. Each visit took 45–90 minutes depending on the size and features of the centre. Centre visits were completed during March–April 2015 prior to commencement of the main PLAYCE study. Auditors were assigned to each centre based on their availability at the centre’s preferred visiting times and the size of the centre to audit, providing a total of 38 paired audits at Time 1 and 2, across the eight participating centres.

To assess inter-rater reliability of the PLAYCE ECEC environmental audit, data from the only two auditors who assessed all eight centres at Time 1 as part of the intra-rater reliability data collection, were combined with data from another pair of auditors. This second pair of auditors conducted the audit at the first 12 ECEC centres that agreed to take part in the main PLAYCE study [28] during September–December 2015, to provide an overall sample of 20 centres for assessing inter-rater reliability. Both sets of auditors followed the same procedure for completing the environmental audit as outlined above.

To assess test-retest reliability of the PLAYCE ECEC educator practice survey, the centre director and educators who cared for children 2–5 years were invited to complete the first survey (Time 1) during the audit team visit, and the second survey (Time 2) two weeks later.

### 2.4. Statistical Analysis

Socio-economic status (SES) of the ECEC services’ location was determined by the Socio-Economic Indexes for Areas (SEIFA) suburb ranking [33] and categorised into tertiles. Size of centres was determined according to the number of approved child places and divided into quartiles—small (≤ 41 places), small/medium (42–57 places), medium/large (58–74 places) and large (≥ 75 places).

For the PLAYCE ECEC environmental audit, individual play equipment item scores were truncated at 20 and the number of types or individual pieces of equipment, features and spaces were totalled. Intra-rater reliability was assessed by comparing the completed audits for each auditor at Time 1 and Time 2, reporting single measure intraclass correlation coefficients (ICCs) using a one-way random absolute model for continuous variables and Cohen’s Kappa with percent agreement for categorical variables. Inter-rater reliability was assessed by comparing completed audits between the two pairs of raters, using the same statistics. Test-retest reliability for the PLAYCE ECEC educator survey was assessed by comparing the responses for each Educator at Time 1 and Time 2. Categorical items and ordinal scales were assessed using a weighted Kappa statistic and percent agreement.

ICCs were rated using cut points of: < 0.40—poor; 0.40 to 0.59—fair; 0.60 to 0.74—good; and 0.75 to 1.00—excellent. Kappa statistics were rated using cut points of: < 0.00—Poor; 0.00–0.20—Slight; 0.21–0.40—Fair; 0.41–0.60—Moderate; 0.61–0.80—Substantial; 0.81–1.00—Almost perfect).

## 3. Results

### 3.1. Sample Characteristics 

Of the eight centres that participated in the environmental audit intra-rater reliability testing, 38% were from the lowest SES tertile, 12% were from the middle and 50% were from the highest. Half of the centres were large in size and 25% were medium-to-large. On average, five auditors visited each centre (range: 3–9), and audits were completed seven days apart.

Of the 20 centres included in the environmental audit inter-rater reliability testing, 50% were from the lowest SES tertile, 15% were from the middle, and 35% were from the highest SES tertile. The majority of the centres were very either small (35%) or large (50%).

A total of 32 educators from seven of the eight centres participating in the environmental audit testing completed the educator practice survey at Time 1 and Time 2. Educators were aged 20–57 years (mean 33 years; SD 11.1), 97% were female and 28% held a diploma or higher degree. Surveys were completed on average 13 days apart (SD 7.13).

### 3.2. Intra- and Inter-Rater Reliability of Play Spaces and Environments for Children’s Physical Activity Early Childhood Education and Care (PLAYCE ECEC) Environmental Audit

Intra-rater reliability ICCs for outdoor equipment, spaces and features were excellent (ICC = 0.80–0.94) (Table 1). The results for indoor equipment, media and spaces were more varied with indoor fixed play equipment the least reliable (ICC = 0.48—fair) and indoor fixed media equipment the most reliable (ICC = 0.77—excellent). Kappa ratings of moderate agreement or above were achieved for more than three quarters of the items. Intra-rater reliability showed slight agreement for the two “condition of outdoor equipment” items, although percent agreement was 79% and 69% for condition of fixed and portable outdoor equipment, respectively.

Inter-rater reliability ICCs for outdoor equipment, spaces and features were good to excellent (ICC = 0.70–0.79) (Table 1). Results for indoor equipment, media and spaces varied from excellent for indoor fixed play equipment (ICC = 0.78) to fair for indoor portable play equipment (ICC = 0.46) and indoor portable media equipment (ICC = 0.47). For categorical items half showed moderate or substantial inter-rater reliability. The items addressing “condition of fixed outdoor equipment”, “outdoor running space” and “outdoor path shape” demonstrated poor or slight agreement (*κ* ≤ 0.20) and percent agreement was 74%, 55% and 42% respectively.

### 3.3. Test-Retest reliability of the PLAYCE ECEC Educator Practice Survey

Kappa statistics across the 57 items ranged from −0.07 to 0.82 and percent agreement ranged from 38% to 94% (Table 2). Using the Kappa rating, more than two thirds of items were rated moderate agreement or above. Due to large imbalances in the distribution of responses, three items recorded negative Kappa statistics—“frequency that educators supervise during physical activity time” (*κ* = −0.04), “duration of TV time per week” (*κ* = −0.03) and “frequency that TV time is used as a reward” (*κ* = −0.07), although percentage agreement scores for these items ranged from 85% to 88%, indicating a high level of consistency.

## 4. Discussion

Reliable measures of environmental attributes are vital for understanding how the ECEC environment influences young children’s physical activity and for addressing inconsistent findings to date. This study evaluated the reliability of the PLAYCE study ECEC environmental audit and educator practice survey, designed to assess the environments that influence young children’s physical activity while at ECEC as part of a socio-ecological approach. The results show that the environmental audit provides excellent intra-rater reliability and good to excellent inter-rater reliability for outdoor equipment, natural features and play spaces. Audit items that consisted of presence or observed counts generally performed better than those that required a quality assessment. Furthermore, the majority of items measuring educators’ physical activity-related practices were rated as moderate agreement and above. The PLAYCE study instruments extend previous tools by including the assessment of ECEC natural features and indoor play spaces, to provide a reliable and comprehensive assessment of the physical, social and policy environment related to physical activity in ECEC.

The PLAYCE study instruments were developed to improve upon previous tools to comprehensively assess the ECEC environment for physical activity in the Australian context [31,32]. The environmental audit differentiates itself from other observational audits by comprehensively assessing both the indoor and outdoor ECEC environment. Physical activity equipment and the size of play space have been included in most instruments using an ecological approach to measure the ECEC physical environment [34,35]. There has been less focus on other elements of the physical environment such as outdoor and indoor design features and spaces for different types of play. While these elements have been associated with physical activity within ECEC settings in individual studies [12], they have not been routinely measured alongside other physical, social and policy environment measures. Furthermore, the two PLAYCE study instruments complement each other by providing an assessment of the presence of physical activity related equipment and spaces, as well as aligned educator practices (e.g., frequency equipment and spaces are available, and used for physical activity). This will allow further exploration of how ECEC physical and social environments may interact to facilitate or inhibit children’s physical activity.

Robust measurement of ECEC environments can also play an important role in monitoring and assessing ECEC quality standards [23]. In Australia, the Children’s Education and Childcare Quality Authority (ACECQA) regularly assess ECEC centres on meeting quality, standards and elements, including ‘children’s health and safety’ and ‘physical environment’. However, these quality area standards, their assessment and ECEC accreditation are not currently aligned to physical environmental factors known to influence young children’s physical activity in ECEC settings. The data collected by the environmental audit and educator practice survey during the PLAYCE study can be used to inform future ACECQA standards and their assessment [28].

This study had several limitations. While written and pictorial descriptors were included to provide greater clarity on what constituted a particular piece of equipment or play space, items assessing the condition of equipment may also have benefited from further description and auditor training as these performed most poorly. Furthermore, large imbalances in the distribution of responses to some items resulted in high levels of percent agreement but low Kappa values. Also, while the study tested three types of reliability, validity of the instruments was not tested. Finally, the PLAYCE study instruments have been implemented in the Australian ECEC context and testing in other countries with differing physical, social and policy environments is recommended. Despite these limitations, the instruments described in this study are both comprehensive and reliable and enhance current methods of environmental data collection in ECECs. The PLAYCE instruments may help with the benchmarking of current environmental standards within ECECs and facilitating further research to identify influences and support the improvement of ECEC environments for young children’s physical activity.

## 5. Conclusions

The ECEC setting provides an important intervention opportunity for increasing physical activity and improving the health and development of young children. The PLAYCE study instruments provide reliable measures of the social, physical and policy environment for physical activity in ECECs, and can help to better understand the factors that influence physical activity and sedentary behaviour in early childhood and resolve current inconsistencies in the literature. The PLAYCE study instruments have been used to assess the ECEC environment and contribute to recommendations on meeting regulatory quality standards for children’s health and the environment for ECEC in Australia.

## Figures and Tables

**Table 1:** Intra—and inter-rater reliability results for the early childhood education and care (ECEC) environmental audit.

Environmental Audit Items	Intra-Rater Reliability (*n* = 38)			Inter-Rater Reliability (*n* = 20)		
	Time 1 Mean (SD)	Time 2 Mean (SD)	ICC (95% CI) / Kappa Statistic (% Agreement)	Rater 1 Mean (SD)	Rater 2 Mean (SD)	ICC (95% CI) / Kappa Statistic (% Agreement)
Outdoor physical activity (PA) equipment						
Fixed play equipment—total no. ^h^	18.87 (15.49)	18.61 (15.15)	0.94 (0.90–0.97)	15.65 (8.95)	15.95 (13.09)	0.70 (0.38–0.87)
Fixed play equipment—condition ^a, i^			0.19 (78.8%)			−0.09 (73.7%)
Portable play equipment—total no. ^h^	67.50 (29.48)	65.80 (31.10)	0.94 (0.88–0.97)	60.60 (24.86)	57.35 (23.28)	0.74 (0.45–0.89)
Portable play equipment—condition ^a, i^			0.15 (68.6%)			0.57 (85.0%)
Outdoor play environment						
Play spaces—types ^i^	3.05 (1.14)	3.08 (1.12)	0.80 (0.66–0.89)	3.15 (1.04)	3.00 (1.03)	0.79 (0.55–0.91)
Running space ^b, j^			0.44 (71.1%)			0.04 (55.0%)
Path shape for wheeled toys ^c, k^			0.69 (81.6%)			0.50 (70.0%)
Path connection to outdoor play space ^d, l^			0.40 (56.8%)			0.20 (42.1%)
Sides of building accessible ^e, m^			0.89 (97.3%)			0.77 (95.0%)
Natural features—types ^i^	7.55 (2.10)	7.39 (1.84)	0.84 (0.71–0.91)	6.60 (1.79)	6.65 (1.95)	0.79 (0.55–0.91)
Indoor PA equipment						
Fixed play equipment—types ^h^	0.61 (0.72)	0.47 (0.70)	0.48 (0.19–0.70)	0.35 (0.59)	0.30 (0.57)	0.78 (0.52-0.90)
Fixed Play equipment—condition ^a. i^			0.76 (93.0%)			Ltd data
Portable play equipment—types ^h^	1.54 (0.87)	1.69 (0.90)	0.59 (0.33–0.77)	1.00 (0.86)	0.70 (0.73)	0.46 (0.05–0.75)
Portable play equipment—condition ^a, i^			0.51 (78.8%)			0.50 (77.8%)
Indoor media equipment						
Fixed media equipment—total no. ^i^	0.45 (1.01)	0.36 (1.05)	0.77 (0.59–0.87)	0.45 (0.76)	0.35 (0.67)	0.61 (0.27-0.82)
Portable media equipment—total no. ^i^	0.82 (0.80)	0.78 (0.68)	0.55 (0.27–0.74)	0.60 (0.88)	0.60 (0.75)	0.47 (0.06–0.75)
Indoor play environment						
Play space—availability ^f, h^			0.60 (0.78.9%)			0.44 (70.0%)
Indoor play space spatial connection to outdoor ^a, n^			1.00 (100%)			0.35 (63.2%)
Indoor play space visual connection to outdoor ^a, n^			0.67 (83.8%)			0.27 (63.2%)
Play spaces—types ^n^	6.18 (1.14)	5.89 (1.47)	0.49 (0.21–0.70)	7.00 (1.17)	6.30 (1.30)	0.60 (0.24–0.82)
Policy environment						
PA policy—presence ^g, l^			0.84 (92.1%)			0.61 (81.3%)
Screen policy—presence ^g, l^			0.86 (94.7%)			0.60 (80.0%)
Sun policy—presence ^g, i^			NA (100%)			NA (100%)

Answer Scales: ^a^ 1 = Very poor, 2 = Poor, 3 = Average, 4 = Good, 5 = Excellent; collapsed to 1–3 / 4–5; ^b^ 1 = Unobstructed with plenty of space for groups games (tag, red rover, etc.), 2 = Some obstruction, but space was adequate for individual play (running, skipping, etc.), 3 = Plenty of space for play, but obstructed with play equipment, 4 = Little running space or completely obstructed; ^c^ 1 = No path, 2 = Straight, 3 = Curved but not looped, 4 = Curved and looped; collapsed to 1 / 2 / 3–4; ^d^ 1 = No path, 2 = Connects to building entrances only, 3 = Connects the building to play areas only, 4 = Connects different play areas to each other only, 5 = Two types of connections, 6 = Three types of connections; collapsed to 1 / 2–4 (1 connection) / 5 (2 connections) / 6 (3 connections); ^e^ collapsed to 1–2 sides / 3–4 sides; ^f^ 1 = Quiet play (not a lot of room for movement e.g., classroom is small or crowded with furniture), 2 = Limited movement/some active play (able to walk, skip, hop, jump around), 3 = All activities (easily able to perform all gross motor activities); ^g^ 1 = Yes, 0 = No;. Item Sources: ^h^ (Ball et al., 2005)—modified; ^i^ New item; ^j^ (Ball et al., 2005); ^k^ (Ward et. al., 2014); ^l^ (Ward et al., 2014)—modified; ^m^ (Olesen et al., 2013); ^n^ (Moore, 2010)—modified.

**Table 2:** Test-retest reliability results for the ECEC educator practice survey.

Educator Practice Survey Items	Kappa Statistic (*κ*)	Percentage Agreement (%)
Educator influences on physical activity (PA) behaviour		
Duration of indoor PA provided to children/day ^a, g^	0.45	40.63
Duration of outdoor PA provided to children/day ^a, g^	0.53	75.00
Duration of educator led PA provided to children/day ^a, g^	0.68	65.63
Frequency of types of indoor PA (4 items) ^b, g^	0.18–0.58	43.75–84.38
Frequency of types of outdoor PA (7 items) ^b, g^	0.18–0.78	53.13–81.75
Frequency educators take away PA as behaviour management ^c, g^	0.47	59.38
Frequency educators supervise during PA time ^c, g^	−0.04	84.38
Frequency educators verbally encourage during PA time ^c, g^	0.21	78.13
Frequency educators join in during PA time ^c, g^	0.47	71.88
Frequency educators incorporate PA into classroom routines, transitions, planned activities ^c, h^	0.65	75.00
Frequency educators talk with children about importance of PA ^c, h^	0.50	71.88
Variety of PA promotional materials (books, posters etc) ^d, h^	0.52	65.63
Frequency of professional development received by educators by topic (4 items) ^e, i^	0.37–0.63	62.50–81.25
Educator influences on sedentary behaviour		
Longest duration children seated at any one-time ^f, g^	0.69	84.38
Duration of television (TV) time/week ^a, g^	−0.03	87.50
Duration of interactive screen time/week ^a, g^	0.78	90.63
Frequency alternative options are offered in place of screen time ^c, h^	0.66	71.88
Frequency TV time is used as a reward ^c, g^	−0.07	84.38
Frequency interactive screen time is used as a reward c, g	0.50	90.63
PA equipment		
Frequency play equipment available during free play time (4 items) ^c, g^	0.45–0.56	50.00–78.13
Frequency new PA equipment is purchased to replace old ^c, i^	0.51	56.25
Indoor and outdoor spaces		
Frequency indoor play areas available for children’s use (9 items) ^b, j^	0.27–0.69	37.50–93.75
Frequency indoor play areas used for educator-led activities (9 items) ^b, j^	0.32–0.63	50.00– 84.38
Frequency doors between indoor and outdoor spaces are open ^c, i^	0.52	62.50
Frequency children are allowed to move freely between indoor and outdoor spaces ^c, i^	0.69	65.63

Answer Scales: ^a^ 1= Less than 30 min, 2 = 30–59 min, 3 = 60–89 min, 4 = 90 min or more; ^b^ 1 = Never, 2 = Less than once a week, 3 = Once a week, 4 = Most days, 5 = Every day; ^c^ 1 = Rarely or never, 2 = Sometimes, 3 = Often, 4 = Always; ^d^ 1 = Few or no materials, 2 = Some materials with limited variety, 3 = A variety of materials, 4 = A large variety of materials with items added or rotated seasonally; ^e^ 1 = Rarely, 2 = 1 time per year, 3 = More than 1 time per year; ^f^ 1 = Less than 15 min, 2 = 15–29 min, 3 = 30–44 min, 4 = 45 min or more; Item Sources: ^g^ (Ward et al., 2014)—modified; ^h^ (Ward et al., 2014); ^i^ New item; ^j^ (Moore, 2010)—modified; ^k^ Range of Kappa values and percent agreement presented for questions with multiple items.
